# Structural and Physiological Analyses of the Alkanesulphonate-Binding Protein (SsuA) of the Citrus Pathogen *Xanthomonas citri*

**DOI:** 10.1371/journal.pone.0080083

**Published:** 2013-11-25

**Authors:** Fabiano Tófoli de Araújo, Victor M. Bolanos-Garcia, Cristiane T. Pereira, Mario Sanches, Elisa E. Oshiro, Rita C. C. Ferreira, Dimitri Y. Chigardze, João Alexandre Gonçalves Barbosa, Luís Carlos de Souza Ferreira, Celso E. Benedetti, Tom L. Blundell, Andrea Balan

**Affiliations:** 1 Departamento de Microbiologia, Universidade de São Paulo, São Paulo, São Paulo, Brazil; 2 Department of Biochemistry, University of Cambridge, Cambridge, United Kingdom; 3 Laboratório Nacional de Biociências, Centro de Pesquisa em Energia e Materiais, Campinas, São Paulo, Brazil; 4 Monte Sinai Hospital, Toronto, Ontario, Canada; 5 Departamento de Genética, Universidade Católica de Brasília, Brasilia, Districto Federal, Brazil; Griffith University, Australia

## Abstract

**Background:**

The uptake of sulphur-containing compounds plays a pivotal role in the physiology of bacteria that live in aerobic soils where organosulfur compounds such as sulphonates and sulphate esters represent more than 95% of the available sulphur. Until now, no information has been available on the uptake of sulphonates by bacterial plant pathogens, particularly those of the *Xanthomonas* genus, which encompasses several pathogenic species. In the present study, we characterised the alkanesulphonate uptake system (Ssu) of *Xanthomonas axonopodis* pv. *citri* 306 strain (*X. citri*), the etiological agent of citrus canker.

**Methodology/Principal Findings:**

A single operon-like gene cluster (*ssuEDACB*) that encodes both the sulphur uptake system and enzymes involved in desulphurisation was detected in the genomes of *X. citri* and of the closely related species. We characterised *X. citri* SsuA protein, a periplasmic alkanesulphonate-binding protein that, together with SsuC and SsuB, defines the alkanesulphonate uptake system. The crystal structure of SsuA bound to MOPS, MES and HEPES, which is herein described for the first time, provides evidence for the importance of a conserved dipole in sulphate group coordination, identifies specific amino acids interacting with the sulphate group and shows the presence of a rather large binding pocket that explains the rather wide range of molecules recognised by the protein. Isolation of an isogenic *ssuA-*knockout derivative of the *X. citri* 306 strain showed that disruption of alkanesulphonate uptake affects both xanthan gum production and generation of canker lesions in sweet orange leaves.

**Conclusions/Significance:**

The present study unravels unique structural and functional features of the *X. citri* SsuA protein and provides the first experimental evidence that an ABC uptake system affects the virulence of this phytopathogen.

## Introduction

All living organisms require sulphur for the biosynthesis of amino acids (cysteine and methionine) and cofactors such as glutathione, coenzyme A and coenzyme M [1]. Bacteria must either obtain these molecules directly from the environment or synthesise them using inorganic (e.g., sulphate) or organic (e.g., sulphonates) sulphur sources [2], [3]. In aerobic soils, the sulphur content is almost entirely represented by sulphonates and sulphate esters of various organic compounds, with inorganic sulphur representing less than 1–5% of the available element [4]. Under such conditions, several bacterial species are known to express proteins required for the uptake of organic sulphur-containing substances such as sulphate esters, sulphamates, sulphonates and alkanesulphonates [1], [5], [6].

In *Escherichia coli*, the uptake of sulphur-containing organic compounds requires the expression of two ATP binding cassette (ABC) transport systems: the Tau system, which is specifically involved in the uptake of taurine, and the Ssu system, which mediates the uptake of other aliphatic sulphonates [5], [7]. The Ssu system is encoded by a single operon encompassing five cistrons, *ssuEADCB,* that encode the three components of the uptake system, which are the periplasmic alkanesulphonate-binding protein (SsuA), the membrane permease protein (SsuC) and the nucleotide-binding protein (SsuB), and two cytoplasmic proteins involved in sulphur release, NAD(P)H flavin mononucleotide oxidoreductase (SsuD) and the FMNH2-dependent sulphonate monooxygenase (SsuE) [7]. Structural analysis of the Ssu uptake system has been restricted to the recent description of the substrate-free *E. coli* SsuA protein [8]. The SsuA protein is similar to other periplasmic ABC transporters in which two globular domains form a cleft in which the ligand binds and from which it is subsequently translocated to the membrane-bound compartment. However, no information is presently available about the structure of the SsuA protein-ligand complex, the interactions of specific amino acid residues with the alkanesulphonate substrates or the molecular features that allow various alkanesulphonate molecules to fit into the protein’s ligand-binding pocket.

In addition to the Ssu system of *E. coli*, functional Ssu uptake systems have also been reported in *Bacillus subtilis* and *Pseudomonas putida*, further supporting the relevant physiological role of this nutrient uptake system in soil-inhabiting bacteria [6], [9]. Despite the apparently relevant physiological role of the Ssu system, no information is available regarding the uptake of organic sulphur-containing compounds by bacterial plant pathogens. Indeed, once absorbed by plants as inorganic sulphate, sulphur is rapidly converted into complex organic molecules such as proteins and sulphonates or to sulphoquinovose in sulpholipids of thylakoid membranes [7]. However, no information regarding the role of aliphatic sulphonate uptake in the growth and virulence of phytopathogens is presently available.

The *Xanthomonas* genus encompasses 27 different bacterial species and over 140 pathovars that interact with more than 400 plant species, including several economically relevant species [10], [11]. X*anthomonas citri*, the causative agent of citrus canker, is capable of infecting all citrus cultivars, although different citrus species may show distinct susceptibility to the disease, as illustrated by the high susceptibility of sweet orange (*Citrus sinensis*) and the lower susceptibility of mandarin species [12]. Canker is one of the most economically damaging diseases of citrus plants; it begins with the epiphytic colonisation of the leaf surface followed by the entrance of the pathogen into leaf tissue through stomata or wounds. Upon entrance into the plant mesophyll, the bacterium induces cell enlargement and multiplication (hyperplasia) followed by a water-soaking phenotype and the formation of blister-like lesions approximately 4 days after infection. The production of highly hygroscopic xanthan gum helps bacteria increase the water adsorption of the plant through the capillary effect from xylem, leading to disruption of the plant epidermis and formation of yellow spongy pustules that become brown and corky with time [13].

In the present study, we investigated for the first time the presence of alkanesulphonate uptake systems in the *Xanthomonas* genus, with particular emphasis on the system encoded by *X. citri*. A single operon-like gene cluster (*ssuEDACB*) encoding both the uptake system and the intracellular enzymes involved in sulphur release was detected in the genome of the *X. citri* 306 strain but not in other *Xanthomonas* species with the exception of the closely related *X. fuscans*. The structure of SsuA in complex with MOPS, MES and HEPES was determined, showing the importance of a conserved dipole at the substrate cleft for sulphate group coordination and the presence of a rather spacious ligand pocket, partially filled with water molecules, that permits the binding of alkanesulphonates of quite different molecular sizes, charges and shapes. We also showed that an active alkanesulphonate uptake system is required for the growth of *X. citri* under sulphate-restricted conditions. Finally, generation of an *ssuA-*knockout mutant of the *X. citri* 306 strain showed that a defective alkanesulphonate uptake system affects both xanthan gum production and the generation of canker lesions in a susceptible citrus host. Altogether, the present work represents the first structural and functional characterisation of an alkanesulphonate uptake system of a bacterial plant pathogen and demonstrates that this nutrient uptake system plays a role in the pathogenesis of *X. citri*.

## Results

### The alkanesulphonate uptake system in *Xanthomonas*

**Figure 1:**
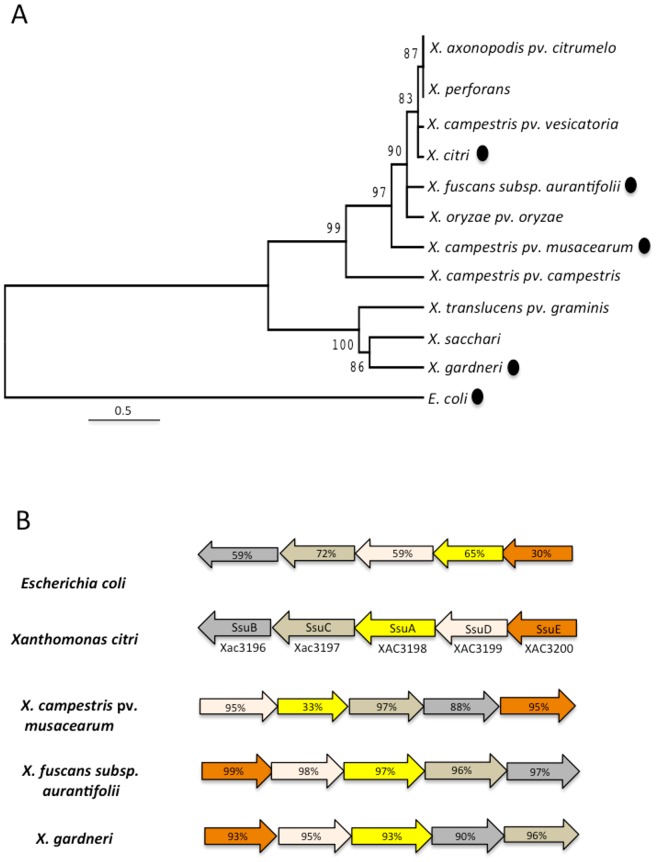
The presence of SsuA in *Xanthomonas* species and the genetic organisation of the s*su* operon in *E. coli* and *X. citri*. (A) Neighbour-joining tree based on 16S rRNA processing protein RimM showing relationships among *Xanthomonas* species and other species encoding SsuA proteins (black balls). Distances were determined using sequences aligned by ClustalW. (B) Genetic organisation of the *ssu* operon in *E. coli, X. citri* and other *Xanthomonas* species in which it was found. The amino acid sequence identities of the orthologues related to *X. citri* proteins are indicated as percentages inside the arrows. Genes are represented by the same colours used for the *X. citri* operon. SsuA: periplasmic-binding protein; SsuB: nucleotide-binding protein; SsuC: ABC transporter permease; SsuD: NAD(P)H-dependent FMN reductase; SsuE: alkanesulphonate monooxygenase FMNH(2)-dependent.

A search of the available *Xanthomonas* genomes for genes involved in the uptake of alkanesulphonates revealed 4 species (*X. citri*, *X. fuscans*, *X. gardneri*, and *X. campestris*) in which a single operon-like gene cluster sharing similarity with *E. coli ssu* genes was found (**Figure 1**). The *X. citri ssu* genes showed a similar genetic organisation to that of the *ssu* operon found in *E. coli* with the exception that *ssuD* and *ssuA* were present in inverted positions in the two species. The fact that the putative functional organisations of these genes have been maintained in two citrus pathogens indicates that the *ssu* genes have a recent evolutionary history in the genus. Further structural and functional analyses of the alkanesulphonate uptake genes concentrated on *X. citri* SsuA, the alkanesulphonate-binding protein. The deduced amino acid sequence of the *X. citri* SsuA protein shared 59% identity with the *E. coli* orthologue, and the *X. citri* SsuA gene was chosen for subsequent cloning, expression and purification of a recombinant form of the protein.

### Interaction of recombinant *X. citri* SsuA with various alkanesulphonate substrates

**Figure 2:**
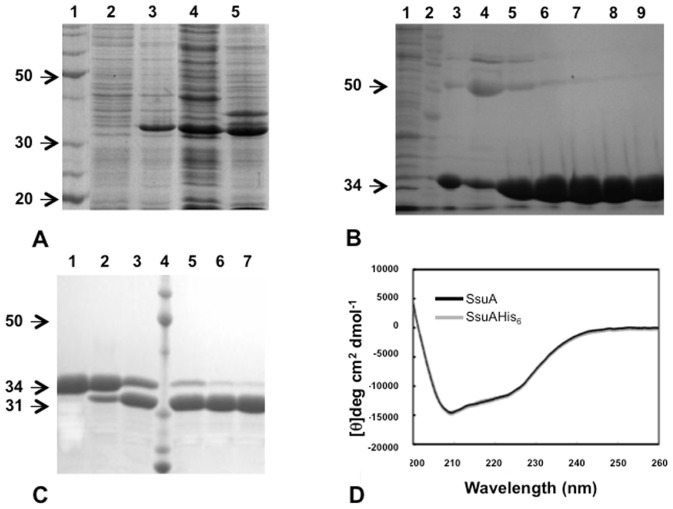
Expression and purification of recombinant *X. citri* SsuA. Production of the folded purified alkanesulfonate-binding protein SsuA of *X. citri*. (A) SsuA expression from *E. coli* BL21(DE3) cells. Lanes: 1) molecular weight markers; 2) whole cell extract of the non-induced strain; 3) whole cell extracts of the strain after induction with IPTG for 2 hs; 4) soluble fraction of the whole cell extract of the induced strain; 5) insoluble fraction of the whole cell extract of the induced strain. (B) Purification of SsuA by immobilized metal affinity chromatography. Lanes: 1) Flow through; 2) molecular weight markers; 3–4) Washing steps with 20 mM imidazol; 5–9) elution fractions using 50 mM to 500 mM imidazol. (C) Cleavage of recombinant SsuA with thrombin for cut-off of the His_6_tag. Lanes: 1) no treated SsuA; 2–3) SsuA incubated with thrombin for 1 and 2 h, respectively; 3) molecular weight markers; 4–6) SsuA digests after incubation for 4, 8 and 16 h, respectively. (D) Circular dichroism spectra of the SsuA with and without the His6tag.

**Figure 3:**
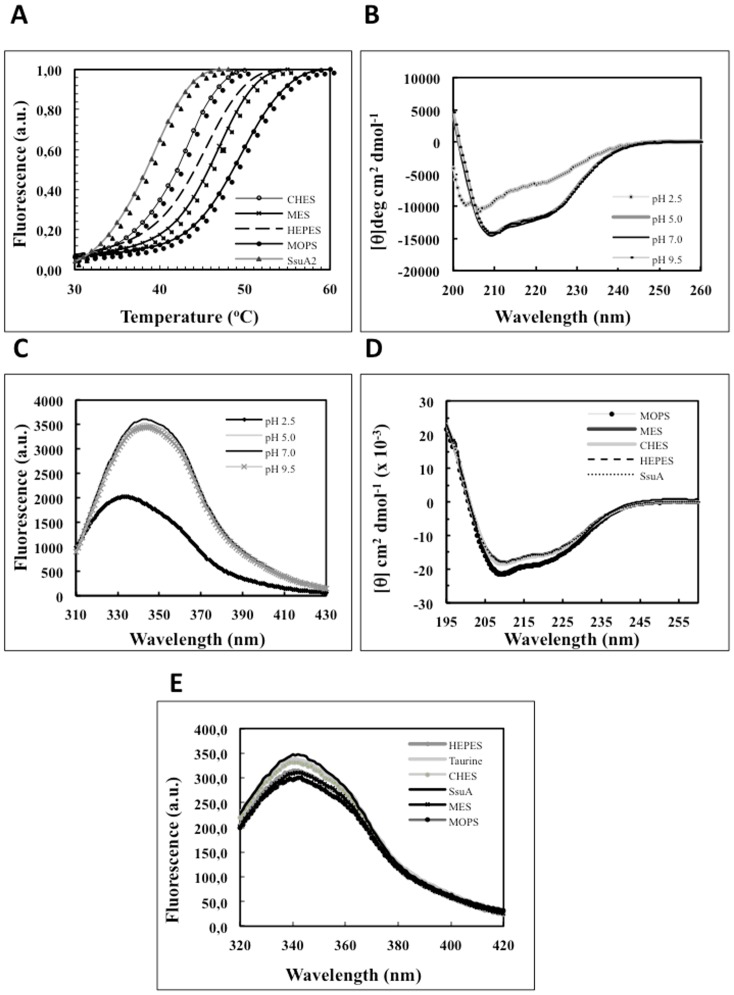
Spectroscopic analysis of *X. citri* SsuA protein in the presence of alkanesulphonates and at different pH values. (A) Thermal shift assay in the presence of various alkanesulphonates (CHES, MOPS, MES, or HEPES). (B) Circular dichroism and (C) fluorescence analyses of the recombinant protein at different pH values. (D) Circular dichroism and (E) intrinsic fluorescence of the recombinant *X. citri* SsuA in the presence of ligands. The stability of the protein at various pH values and in the presence of various aliphatic sulphonates was monitored by following the intrinsic fluorescence of the tryptophan residues using an Aminco BOWMAN series 2 spectrofluorometer.

The recombinant *X. citri* SsuA protein was expressed as a soluble cytosolic protein genetically fused with a histidine tag and a thrombin cleavage site at the N-terminal end. Maximum soluble protein yields (∼80 mg/L) were achieved after expression under optimum inducing conditions. The protein was purified by single-step affinity chromatography and subsequently cleaved with thrombin to remove the vector-encoded histidine (**Figure 2**). The purified recombinant protein remained soluble and stable at high concentrations (6–12 mg/mL) even after extended storage at –20°C. Thermal shift experiments showed increased stability of recombinant *X. citri* SsuA in the presence of MOPS, MES, CHES, or HEPES but not in the presence of taurine, sulphate, thiosulphate or hydroxylamine (**Figure 3A**). These results indicate that the recombinant protein binds specifically to alkanesulphonate ligands. The *Tm* of SsuA varied from 39.5°C for the unbound form to 43.5°C (CHES), 45.8°C (HEPES), 47.1°C (MES) and 51°C (MOPS) for the putative ligand-bound forms. CD and fluorescence analyses showed that the recombinant SsuA was stable at pH values of 5, 7 and 9 (**Figure 3B and 3C**) and that it undergoes small changes in secondary structure content upon ligand binding (**Figure 3D and 3E**). The quenching of fluorescence observed after the addition of alkanesulphonates corroborated the CD results and suggested the possible presence of tryptophan residues close to the ligand-binding pocket (**Figure 3E**). Taken together, these results show that recombinant SsuA undergoes conformational changes on binding to alkanesulphonates.

### The structure of ligand-bound *X. citri* SsuA

**Figure 4:**
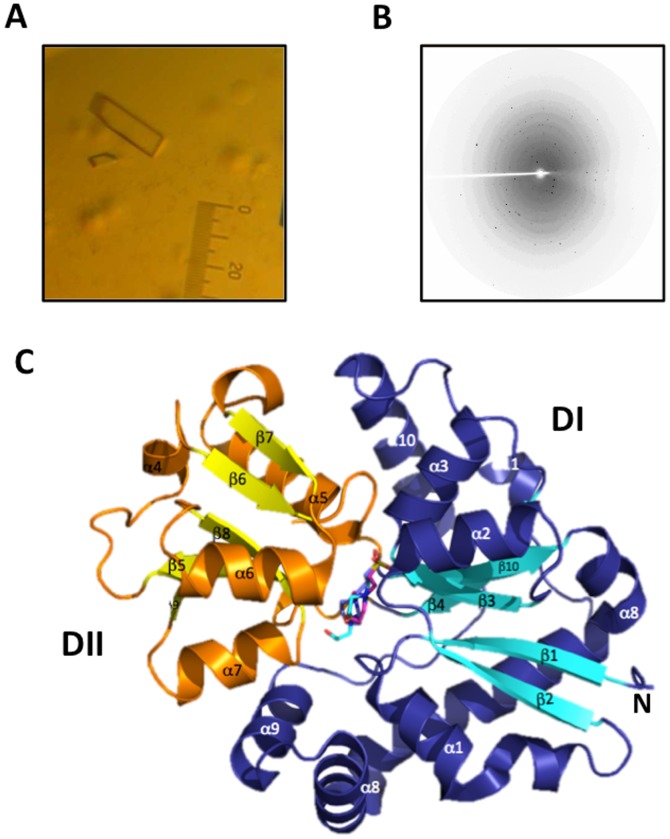
Crystallisation, X-ray diffraction pattern and determination of the tertiary structure of *X. citri* SsuA. (A) SsuA crystals grown in 0.1 M HEPES, pH 7.3, 1.5 M ammonium sulphate and 0.1 NaCl using 6 mg/ml of protein in 20 mM Tris buffer, pH 7.0, containing 50 mM NaCl. (B) Diffraction pattern of SsuA crystal at 2.0 Å resolution. Data were collected at the D03B-MX1 beam line Brazilian Synchrotron Light Laboratory (LNLS) using 1.433 Å radiation and recorded on a *MARCCD*165 detector (oscillation data with Δφ = 1.0^o^). (C) Cartoon illustration of the overall structure of SsuA bound to HEPES, MOPS and MES (stick) showing the alpha-beta structures of domains I (deep blue and cyan) and II (orange and yellow). The N-terminus is shown in domain I.

SsuA crystallised under a variety of conditions in which ammonium sulphate and alkanesulphonates (HEPES and MES) were present (**Table S1**). Initial crystallisation trials of the ligand-bound form of recombinant SsuA resulted in needle-like crystals that grew after one week in the presence of ammonium sulphate, MES and PEG. Refinement of these conditions produced better crystals in the absence of PEG and the presence of sodium chloride. The best diffraction patterns (1.7 and 1.9 Å) were obtained from crystals grown in 0.1 M NaCl, 1.6 M ammonium sulphate, and 0.1 M HEPES, pH 7.5 (**Figures 4A and 4B**). These crystals showed symmetry and systematic absence of the orthorhombic space group P2_1_. **Table 1** summarises the data-collection statistics. The Matthews coefficient was calculated to be 1.67, and a solvent content of 26%, corresponding to 1 molecule in the asymmetric unit, was calculated. The SsuA crystal structure revealed the characteristic folding of periplasmic-binding proteins, which consists of an α/β sandwich pattern organised into two domains (I and II) separated by a cleft at the binding site, with the ligand remaining hidden inside the pocket (**Figure 4C**). The N- and C-termini are present in domain I, which is connected by a hinge (Pro^107^ to Thr^109^ and Gly^204^ to Gly^211^) to domain II. The tertiary structures of SsuA bound to MOPS and MES (PDB codes 3KSJ and 3KSX, respectively) were generated by molecular replacement using the structural coordinates of SsuA bound to HEPES (PDB code 3E4R). All crystal structures presented one molecule in the asymmetric unit. The refinement statistics, including statistics from the processing of the collected data and structure refinement, are presented in **Table 1**.

**Table 1:** Data processing statistics of the *X. citri* SsuA crystals and refinement data from its structures.

Crystal	Native SsuA+HEPES	NaI derivative	CsCl_3_ derivative	SsuA+MOPS	SsuA+MES
*X-ray diffraction data*					
Wavelength (Å)	1.433	1.433	1.433	1.46	1.46
Space group	P2_1_	P2_1_	P2_1_	P2_1_	P2_1_
Unit cell parameters	a = 30.66	a = 30.68	a = 30.68	a = 30.98	a = 30.87
	b = 85.50	b = 85.18	b = 85.18	b = 86.21	b = 86.36
	c = 46.83	c = 46.89	c = 46.90	c = 46.73	c = 46.45
	α = 90.00	α = 90.00	α = 90.00	α = 90.00	α = 90.00
	β = 98.08	β = 98.20	β = 98.20	β = 97.76	β = 97.46
	γ = 90.00	γ = 90.00	γ = 90.00	γ = 90.00	γ = 90.00
Resolution range (Å)	30.00–2.00	30.00–2.30	30.00–2.38	26.00–1.70	26.00–1.99
Rsym (%)	0.062 (0.162)	0.062 (0.162)	0.062 (0.162)	0.09 (0.23)	0.11(0.40)
Completeness (%)	99.3 (99.7)	97.9 (95.7)	98.0 (94.7)	97.9(95.7)	95.1(80.5)
Redundancy	4.3	4.0	4.0	5.8	3.4
<I/sigma>	23.88 (7.48)	13.98 (3.48)	14.18 (3.48)	18.3(3.0)	10.4(2.1)
Mosaicity (deg)	0.456	0.771	0.771	0.500	0.912
Wilson Plot B-factor (Å^2^)	14.6			22.67	41.04
***Refinement and model quality***					
Resolution range (Å)	46.37 – 2.01			26.14–1.70	24.09–1.99
Number of reflections: work/test	15046			21275	14798
Rvalue (%)	0.145			0.162	0.184
Rfree (%)	0.220			0.213	0.256
Overall mean B-factor (Å^2^)	13.96			21.0	38.02

Values in parenthesis correspond to data regarding the last resolution shell.

Footnote: ^a^ R-factor  =  Σ |F_o_(h) – F_c_(h)|/Σ F_o_(h), where F_o_(h) and F_c_(h) are observed and calculated amplitudes for reflection h. R-free is calculated by the same equation using 5 % of the data, chosen randomly and omitted from the refinement.

### The SsuA ligand-binding pocket and interactions with alkanesulphonates

**Figure 5:**
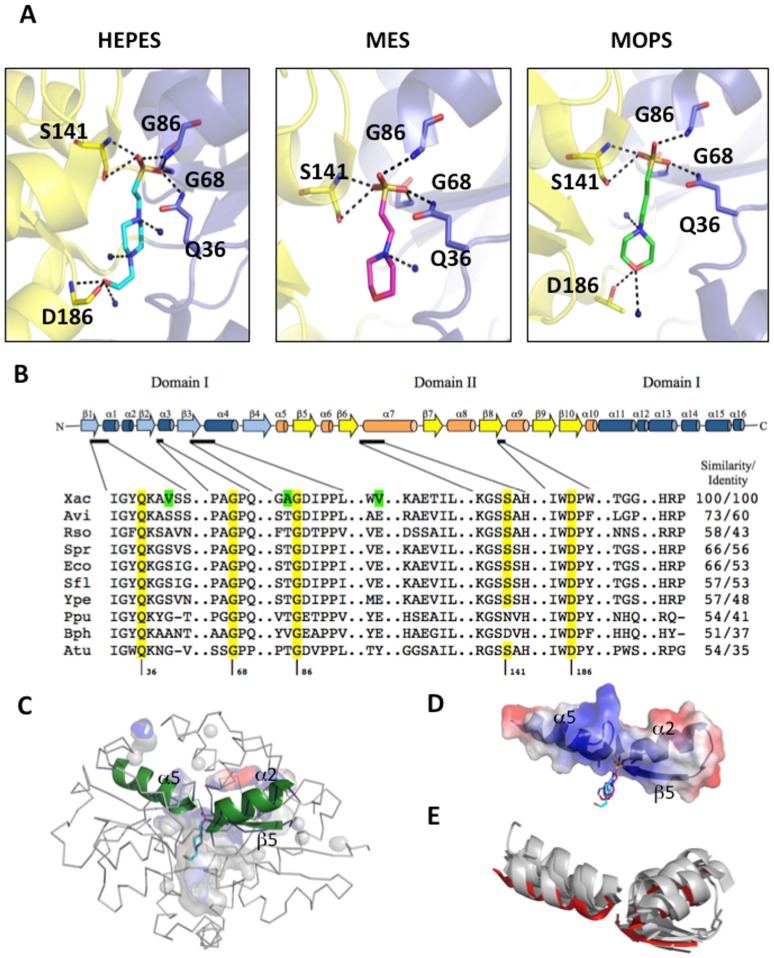
Ligand-binding site and interactions of the *X. citri* SsuA protein. (A) Ligand interactions of SsuA and HEPES, MES and MOPS. Domains I and II are coloured in blue and yellow, respectively; the residues involved in the ligand interaction, as well as the ligands themselves, are shown as sticks. (B) SsuA topology and conservation of the ligand-binding sites in different orthologues. The residues that form the pocket and those that interact with ligands are marked in clear and dark grey, respectively. The residues marked in green are unique to *X. citri* SsuA. The numbering follows the *X. citri* SsuA sequence. Xac: *X. citri* (GI: 21243924); Avi: *Azotobacter vinelandii* (GI: 67153714); Spr: *Serratia proteomaculans* (GI: 5604713); Eco: *E. coli* (GI: 90111189); Sfl: *Shigella flexneri* (GI: 161486517); Ype: *Yersinia pestis* (GI: 22124165); Rso: *Ralstonia solanaraceum* (GI: 207723295); Ppu: *Pseudomonas putida* (GI: 167031279); Bph: *Burkholderia phytofirmans* (GI: 187921640); and Atu: *Agrobacterium tumefaciens* (GI: 159184964). (C) Structure of SsuA in ribbon diagram showing the positioning of the two helices and the beta-sheet (forest cartoon) that form the dipole. The ligand-binding pocket and HEPES inside are shown, respectively, in transparent surface and stick. (D) Electrostatic potential at the surface of the two helices (α2 and α5) and the beta-strand (β3) that contain the residues for dipole formation and sulphate group coordination. (E) Structural superposition of secondary structures of periplasmic-binding proteins (grey) with dipoles similar to that found in the SsuA structure (shown in red). The superposed helices and β-strands belong to the *E. coli* aliphatic sulphonate-binding protein (PDB 2X26), *Sinechocystis* sp. 6856 nitrate-binding protein (PDB 2G29), *E. coli* phosphate-binding protein (PDB 1IXH), *Thermus thermophilus* glutamate/glutamine-binding protein (PDB 1US5) and *X. citri* molybdate-binding protein (PDB 2H5Y).

The crystal structure of SsuA bound to each of the three different alkanesulphonates tested showed that ligand binding is stabilised primarily through a range of polar interactions between the sulphonic acid oxygen atoms and the NH groups of main-chain peptide hydrogen bonds (Gly^68^, Gly^86^, Ser^141^), one side chain NH group of Gln^36^ and one hydroxyl group of Ser^141^ (**Figure 5A** and **Table S2**). Water molecules are responsible for the stability of the alkane chains. Residues in the pocket that interacts with the alkanesulphonates are highly conserved among orthologues from phytopathogens, plant-associated and soil bacteria, and enterobacteria (**Figure 5B**). Some residues exclusively found in *X. citri* SsuA confer a more apolar local environment than that observed in the other bacterial orthologues (**Figure 5B**, green colour).

The SsuA ligand-binding pocket has a volume of 1635+-26 Å^3^ and an area of 2951+-12 Å^2^, but only 14% of the volume is occupied by the ligand molecule (**Figure 5C**). The large binding pocket and the participation of at least 12 hydrophobic residues in forming this site reveal an adaptation that could explain the binding of a rather wide range of alkanesulphonate molecules of different sizes and shapes by this protein. Corroborating the intrinsic fluorescence data, W^185^ faced the binding pocket, stabilising the alkane chain. Interestingly, all of the residues responsible for coordination of the sulphate (Gln^36^, Gly^68/86^ and Ser^141^) belong to two helices and one strand (α2, α5 and β3) (**Figure 5C** **,** in green); together, these residues define a positively charged cluster (**Figure 5D**). A similar structural organisation is found in other periplasmic ion-binding proteins such as the *E. coli* aliphatic sulphonate-binding protein, the *Sinechocystis* sp nitrate-binding protein, the *E. coli* phosphate-binding protein, the *Thermus thermophilus* glutamate/glutamine-binding protein and the *X. citri* molybdate-binding protein (**Figure 5E**).

### Functional roles of *X. citri* SsuA

**Figure 6:**
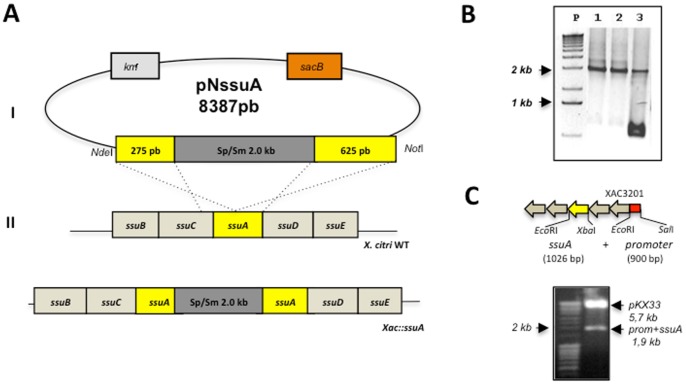
Construction of the *X. citri ssuA*-deleted mutant (*Xac::ssuA*) and complemented strain (*Xac::ssuAc*). (A) Chromosomal deletion of the *ssuA* gene was obtained after electroporation of the suicide pNssuA plasmid into the *X. citri* 306 strain. (I) The first step in the construction of the *X. citri* mutant was the insertion of a 2-kb fragment encoding resistance to spectinomycin and streptomycin into the *KpnI* site of the *ssuA* gene, originating within the pNssuA (8,152 bp) vector. (II) After transformation of wild-type *X. citri* with pNssuA, a double recombination event generated the *Xac::ssuA* mutant strain (III), which was screened by selection of cells resistant to both spectinomycin and sucrose. (B) PCR amplification of *ssuA* genes of selected *X. citri* colonies using primers FssuA2Nde28a and RssuA2Hind28a. Samples: P, molecular weight markers; 1–3, colonies selected for resistance to spectinomycin and sucrose. The presence of a single 2-kb band indicates a successful gene replacement event (samples 1 and 2), while amplification of two bands of 1 kb and 2 kb in size indicates the presence of the chromosomal wild-type gene and a copy of the mutated *ssuA* gene (sample 3). (C) Strategy of cloning and digestion analysis of the pKX33-p*ssuA* plasmid. The localization of *ssuA* gene and the promoter region in the *ssu* operon are evidenced in yellow and red colours, respectively. A band of 1,926 bp was generated after cleavage of the pKX33-p*ssuA* plasmid with *Sal*I and *Xba*I restriction enzymes.

**Figure 7:**
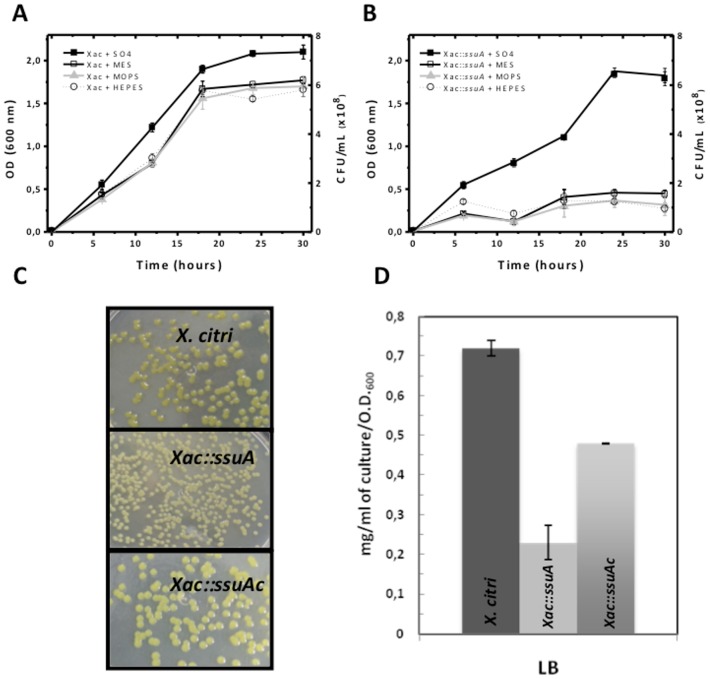
Lack of SsuA affects *in vitro* growth and xanthan gum production by *X. citri*. Growth curve of *X. citri* wild type (A) and the *Xac::ssuA* mutant (B) in M9 media supplemented with sulphate or different alkanesulphonate sources. Samples were taken every 2 h for measuring the growth of the samples. (C) The *Xac::ssuA* mutant shows altered colony morphology after growth at 30°C in LB plates. The strain recovered the normal colony morphology after complementation with the *ssuA* gene. (D) Production of xanthan gum by the parental and *ssuA* mutant strain after 24 h of growth in LB broth. Complementation with pKX33-p*ssuA* restored the reduced xantham gum production observed in the *Xac::ssuA* mutant.

**Figure 8:**
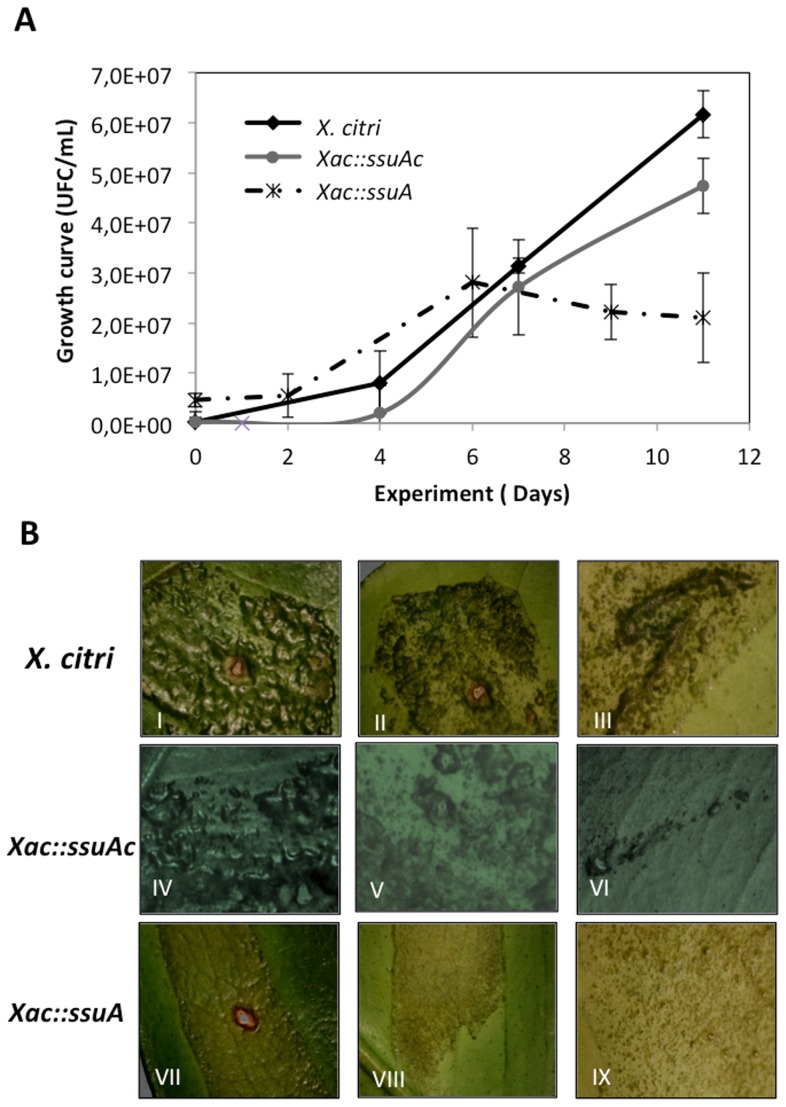
Altered in vivo growth behaviour and leaf lesion formation by the *X. citri ΔssuA* mutant in the *C. sinensis* plant host. (A) Growth curves of *X. citri* wild-type, the isogenic *Xac::ssuA* mutant and the complementary strain (*Xac::ssuAc*) on leaves of *C. sinensis* (susceptible sweet orange cultivar Baia) during an 11-day period after inoculation of 7×10^6^ CFU. The data represent the means of three independently performed experiments. (B) A detailed view of the canker pustules and leaf lesions 14 days after infection. Photographs of the upper surfaces (I, IV and VII) and the undersides (II, V and VIII) of the leaves were taken and enlarged 20- or 30-fold (III, VI and IX), respectively.

To evaluate the physiological role of SsuA during in vitro and in vivo growth of *X. citri*, a *ssuA* knockout strain (*Xac::ssuA*) was generated by site-specific mutagenesis (**Figure 6A and B**). A complementary strain (*Xac::ssuAc*) was generated by transformation of *Xac::ssuA* with the pKX33-p*ssuA* vector, which encoded the full length *ssuA* gene under control of the native *ssu* promoter, giving raise to the *Xac::ssuAc* strain (**Figure 6C**). The isogenic *X. citri ssuA-*deleted strain was unable to grow in minimal medium containing alkanesulphonates (HEPES, MOPS, or MES) as the sole sulphur source but grew well in the presence of sulphate (**Figures 7A and 7B**). This result indicated that the *ssu* operon is functional and is required for alkanesulphonate uptake in *X. citri*. In addition, the *Xac::ssuA* mutant formed small bright yellow colonies after growth in LB plates for 24 h (**Figure 7C**). In both situations the altered phenotypes were reverted in the *Xac::ssuAc* strain (**Figure 7C**). The altered colony morphology of the *Xac::ssuA* strain suggests a decrease in xanthan gum production, a feature previously observed by other groups [14], [15], [16]. Indeed, we determined that xanthan gum production by the *Xac::ssuA* strain during growth in LB medium was at least three-fold lower than that of the parental strain (**Figure 7D**). Complementation with the pKX33-p*ssuA* restored in a great extent production the colony morphology and production of xantham gum by the *Xac::ssuA* strain (**Figure 7D**). To evaluate the effects of alkanesulphonate uptake on the pathogenicity of *X. citri*, we monitored the behaviour of the wild-type, the *Xac::ssuA* **and** ***Xac::ssuAc*** strains after in vivo inoculation into a susceptible citrus host (*C. sinensis*). As shown in **Figure 8A**, the *Xac::ssuA* strain showed defective growth in leaf tissues 6 days after inoculation in comparison with the parental and complemented strains. In addition, the leaf lesions formed by the *Xac::ssuA* strain were smaller than those formed by the parental strain (data not shown). Leaves infected with the *X. citri* 306 strain showed typical blister-like lesions characteristic of the hyperplasia and the water-soaking phenotypes (**Figure 8B, I** to III). Complementation of the *Xac::ssuA* with pKX33-p*ssuA* restored the canker lesions symptoms to the wild-type levels (**Figure 8B**, IV to VI). In contrast, much reduced lesions were observed in leaves inoculated with the *Xac::ssuA* strain, and the lesions that formed were not blister-like (**Figure 8B**, VII to IX). Collectively, these results indicate that the alkanesulphonate uptake system also affects the in vivo behaviour of *X. citri* in *C. sinensis* plants.

## Discussion

Although sulphonates and sulphur-containing organic compounds represent the most abundant sulphur source in most soils, there are no data regarding the role of alkanesulphonate uptake in the behaviour of plant pathogens, particularly those belonging to the *Xanthomonas* genus. In this work, we showed that a complete set of genes required for the uptake and metabolism of alkanesulphonates is found in the genomes of *X. citri* and *X. fuscans,* two closely related citrus pathogens. We expressed and purified a recombinant form of *X. citri* SsuA that specifically binds to various alkanesulphonates but not to sulphate, sulphate esters or a number of other sulphur-containing compounds. Using the recombinant protein, we solved for the first time the three-dimensional structure of a bacterial SsuA protein bound to three different alkanesulphonates (MES, HEPES and MOS). Determination of the crystal structure of the protein permitted the identification of the SsuA protein domains that interact with alkanesulphonate ligands and of the amino acid residues that coordinate these ligands within the ligand-binding pocket of the protein. The rather large binding pocket and the presence of several water molecules clearly accounts for how *X. citri* SsuA accommodates different alkanesulphonates molecules. Finally, we presented evidence that the Ssu system is functional in the *X. citri* 306 strain. Using an isogenic mutant carrying a knockout copy of the *ssuA* gene generated using gene replacement techniques we showed that the Ssu system affects both the in vitro and in vivo behaviour of the strain. In addition, complementation of the *ssuA* mutant strain with a plasmid encoding the wild type gene corrected the altered in vitro and in vivo phenotypes including the capability of the strain to induce disease symptoms in a susceptible citrus host. The present study offers new and relevant information regarding the structural and functional aspects of the alkanesulphonate uptake system of *X. citri* and raises interesting questions regarding the metabolism of alkanesulphonates in the physiology and pathogenicity of this economically relevant phytopathogen.

The search for *ssu* genes homologous to those found in *E. coli* and other bacterial species revealed that, in the *Xanthomonas* genus, only *X. citri, X. fuscans, X. campestris* pv. *musacearum* and *X. gardneri* carry a complete set of *ssu* genes including the ABC transporter and enzymes involved in sulphonate desulphurisation (*ssuD* and *ssuE*). This finding contrasts with the widespread occurrence of functional genes involved with the uptake and metabolism of alkanesulphonates in many bacterial genera such as *Bacillus*, *Pseudomonas*, *Ralstonia*, *Burkeholderia*, *Shingomonas* and *Nostoc*; these bacteria are present in soil and live in environments in which sulphate esters and carbon-bonded sulphur (sulphonates or amino acid sulphur) represent most of the available sulphur [17], [1]. The paucity of *ssu* genes in *Xanthomonas* species suggests that these genes have been recently acquired in the evolutionary history of the genus, most likely by a horizontal gene transfer event. This finding also raises questions about the role of alkanesulphonate uptake in the physiology of the species in which these genes are found.

The remainder of our study focused on the investigation of the Ssu uptake system in *X. citri*, the most economically relevant citrus pathogen. For that purpose, we determined the crystal structure and characterised the physiological role of the SsuA protein, the periplasmic component that confers specificity and affinity to the uptake system [1]. As a first step, we generated a recombinant form of *X. citri* SsuA that retained alkanesulphonate-binding properties similar to those expected for the native protein. As experimentally demonstrated, the recombinant protein exhibited changes in secondary structure and increased thermal stability after specific binding to MOPS, MES, CHES and HEPES but not after exposure to taurine, sulphate, thiosulphate or hydroxylamine. These results indicate that the *X. citri* Ssu system is apparently specific for alkanesulphonates, as previously reported for Ssu orthologues found in *P. putida*, *E. coli* and *B. subtilis* [18], [7], [6].

Previous description of the crystal structure of Ssu components has been restricted to the unbound form of *E. coli* SsuA [8]. The availability of large amounts of recombinant *X. citri* SsuA capable of binding to various alkanesulphonates permitted us to solve for the first time the crystal structure of a bacterial SsuA protein in complex with HEPES, MOPS and MES. The size of the ligand cavity and the presence of 12 hydrophobic residues within it reveal an adaptation of the SsuA protein for interaction with long-chain alkane groups. Similarly relevant is the presence of water molecules in the binding pocket, which may offer flexibility to accommodate the ligands. In agreement with the results of spectroscopic assays, the crystal structure results indicate that SsuA-ligand interactions involve conformational changes associated with movement of the protein domains. According to the three-dimensional structure of SsuA, W^185^ is the most probable residue involved in the observed quenching of fluorescence after ligand binding because it is in close contact with the bound alkanesulphonate and stabilises the aromatic ring of the apolar chains of the molecule. The higher thermal stability evidenced when SsuA is bound to MOPS is due to the presence of shorter hydrogen bonds with the residues belonging to the dipole. Indeed, we showed that alkanesulphonate stabilisation of SsuA is centred in residues that are conserved in different SsuA orthologues, primarily those that form a positive dipole that specifically attracts and interacts with the sulphate group of the alkanesulphonate molecule [19]–[21]. The mechanism of binding and the very positive electrostatic potential of the entrance of the binding pocket suggest that the ligand is attracted to the protein by the sulphate group and then stabilised inside the pocket by the water molecules and the apolar environment. The *X. citri* SsuA structure reported here represents the first report of a bacterial SsuA orthologue bound to different alkanesulphonates and provides a reference for future studies of alkanesulphonate ABC transporters in different bacterial species.

The results obtained in vitro showed that a bacterial strain carrying an inactive copy of the *ssuA* gene failed to grow in the presence of alkanesulphonates such as HEPES, MOPS and MES under inorganic sulphate starvation conditions. This observation indicates that alkanesulphonate uptake is active in *X. citri* and that it may be required under environmental conditions in which the availability of inorganic sulphate is restricted. Xenobiotic and naturally occurring sulphonates, including taurine, isethionate, cysteic acid, methanesulphonate, and several undefined sulphonates, make up a large part of the sulphur present in soil humus and marine sediments [2], [17]. In plants, in addition to sulphonolipids found in thylakoid membranes, a variety of S-containing secondary metabolites that often play important roles in defence against pathogens are synthesised [22]. Our results demonstrated that disruption of the *ssuA* gene resulted in an attenuated phenotype following infection of a susceptible citrus host (*C. sinensis*). This phenotype was attributable at least in part to the reduced production of xanthan gum, which is required for the water soaking phenotype and for the plant’s resistance to various forms of environmental stress [23], [24], [25]. The biosynthesis of xanthan gum involves the coordinate expression of 12 genes that are part of the *gumB-gumM* operon [25], [26]. Twelve of these genes encode proteins with 4 or more cysteine residues. A reduction in the sulphur supply after disruption of the *ssuA* gene may have affected the synthesis of cysteine-enriched proteins and, thus, indirectly reduced xanthan gum production.

Although the precise role of alkanesulphonates during the in vivo growth of *X. citri* is not presently known, our results indicate that alkanesulphonates may have a previously unsuspected relevance to plant-bacterial interactions. Future studies directed at achieving a better understanding of the nutritional and physiological roles of alkanesulphonates in *X. citri* are warranted.

## Methods

### Bacterial strains, plasmids and culture conditions

The *X. citri* 306 strain and the *E. coli* DH5α and BL21 strains were grown in LB medium [27] at 30°C and 37°C, respectively. The *X. citri* 306 strain, the isogenic *ssuA-*knockout mutant and complemented strain *Xac::ssuAc* were also grown in M9 minimal medium [27] and sulphate-free M9 minimal medium supplemented with sulphate or alkanesulphonates as described in the Results section at 28°C under aerobic conditions. When required, kanamycin (25 or 50 µg/ml), spectinomycin (50 µg/ml), and/or ampicillin (100 µg/ml) were added to selective media.

**Sequence analyses.** The amino acid and corresponding nucleotide sequences of the *X. citri ssuA* gene (gi|21243924|ref| NP_643506.1) as well as the *ssuA* orthologue sequences used in this work were obtained from the National Center of Biotechnology Information (http://www.ncbi.nlm.nih.gov). The *Xanthomonas* species phylogeny tree was based on the 16S RNA processing protein RimM accession numbers: *X. axonopodis* pv. *citri* str. 306 (gi: 21107448), *X. campestris* pv. *vesicatoria* str. 85-10 (gi: 78035329), *X. translucens* pv. *graminis* ART-Xtg29 (gi: 424790967), *X. campestris* pv. *campestris* str. ATCC 33913 (gi: 21112243), *X. campestris* pv. *musacearum* NCPPB 4381 (gi: 289670358), *X. axonopodis* pv. *citrumelo* F1 (gi: 346724183), *X. sacchari* NCPPB 4393 (gi: 380511630), *X. albilineans* GPE PC73 (gi: 285017747), *X. perforans* 91-118 (gi: 325924837), *X. fuscans* subsp. *aurantifolii* str. ICPB 11122 (gi: 294626998), *X. gardneri* ATCC 19865 (gi: 325919892), and *E. coli* serotype O157:H7 (gi: 209157890). The proteins that shared high sequence identity with the components of the Ssu transporter are the following: *X. fuscans* subsp. *aurantifolii* nitrate transport protein (ZP_06732183), *X. fuscans* subsp. *aurantifolii* aliphatic sulphonates ATP-binding protein (ZP_06732181), *X. fuscans* subsp. *aurantifolii* ABC transporter permease (ZP_06703616), *X. fuscans* subsp. *aurantifolii, X. fuscans* subsp. *aurantifolii* oxidoreductase (ZP_06703614), *X. fuscans* subsp. *aurantifolii* nitrilotriacetate monooxygenase component A (ZP_06703613.1), *X. campestris* pv. *musacearum* alkanesulphonate transporter substrate-binding (ZP_06488164.1), *X. campestris* pv. *musacearum* ABC transporter ATP-binding subunit (ZP_06488166.1), *X. campestris* pv. *musacearum* ABC transporter permease (ZP_06488165), *X. campestris* pv. *musacearum* oxidoreductase (ZP_06488163.1), *X. campestris* pv. *musacearum* nitrilotriacetate monooxygenase component A (ZP_06488162.1), *X. gardneri* aliphatic sulphonates binding protein (ZP_08184321.1), *X. gardneri* nitrate/sulphonate/bicarbonate ATPase (ZP_08184859.1), *X. gardneri* nitrate/sulphonate/bicarbonate permease (ZP_08184860.1), *X. gardneri* oxidoreductase (ZP_08184320.1), and *X. gardneri* flavin-dependent oxidoreductase (ZP_08184319). Sequence alignments were performed using ClustalW2 [28] at the European Bioinformatics Institute (http://www.ebi.ac.uk/). The phylogenetic tree was accessed by the neighbour-joining method [29], and bootstrap values were obtained from 1,000 duplicates using the MEGA (Molecular Evolutionary Genetic Analysis) package, version 5 [30]. Coordinates and structure factors were deposited in the RCSB Protein Data Bank with PDB codes 3E4R (SsuA + HEPES), 3KSX (SsuA + MOPS) and 3KSJ (SsuA + MES).

**Cloning of the gene encoding** ***X. citri*** **SsuA protein**. All gene cloning steps were carried out with the *E. coli* DH5α strain; expression of the recombinant SsuA protein was carried out with the *E. coli* BL21 (DE3) strain (Novagen). The DNA fragment with the *X. citri ssuA* gene sequence, encoding the mature protein without the first 126 base pairs (42 amino acids) corresponding to the signal peptide, was amplified by PCR (forward primer 5′ gCgCATATggCCgAgCCggCgCA 3′; reverse primer 5′ gCgAAgCTTTCATTTgCTCAC 3′) with Platinum High Fidelity Taq polymerase (Invitrogen) under standard amplification conditions. The amplicon, corresponding to 972 base pairs, was cloned into the vector pGEM T-Easy (Promega) and subcloned into the vector pET28a (Novagen) for expression in *E. coli* BL21 (DE3).

**Expression and purification of recombinant** ***X. citri*** **SsuA.** Cultures of the recombinant *E. coli* BL21 (DE3) strain transformed with pETSsuA were prepared aerobically in LB medium supplemented with 50 µg/mL kanamycin until mid-log phase (OD*_600_* 0.5–0.6); IPTG was then added to a final concentration of 0.1 mM. The cultures were induced aerobically (200 rpm) for 2.5 h at 28°C. Cells were collected by centrifugation at 8,000 *g* for 15 min at 4°C and stored at –20°C for approximately 16 h before lysis. The cell pellets were suspended in buffer 1 (50 mM sodium phosphate buffer, pH 7.2, containing 100 mM NaCl, 5% glycerol and 20 mM imidazole) and incubated with lysozyme (final concentration of 100 µg/mL) and PMSF (1 mM) for 1 h in an ice bath. The cells were sonically disrupted in a Branson Digital Sonifier (Model 450), and soluble fractions were separated from the non-soluble material by centrifugation at 16,000 *g* for 30 min at 4°C. The recombinant SsuA protein was purified from the soluble fraction by immobilised metal affinity chromatography using a HistrapHP column (GE Healthcare) according to the manufacturer’s instructions. The charged resin was washed with buffer 1 (30 bed volumes) followed by step gradient elution with buffers containing increasing concentrations of imidazole (50–500 mM). The eluted fractions were dialysed once against 20 mM Tris-HCl at pH 7 containing 50 mM NaCl and concentrated with Ultrafree MWCO 10,000 centrifugal filters (Amicon Millipore) to a final concentration of 6 mg/mL. The eluted fractions were analysed by SDS-PAGE using 12% acrylamide gels and stained with Coomassie Blue. Protein concentration was determined spectrophotometrically using the Edelhoch method [31]. Recombinant SsuA without the His6-tag was obtained by cleavage of the purified protein with thrombin (Sigma Aldrich, USA) at room temperature. Samples of 20 mg protein were incubated for 2 h with 10 units thrombin in 50 mM Tris, pH 7.5, 10 mM NaCl and 1 mM DTT.

**Spectroscopic analyses of the recombinant SsuA protein.** The stability of the protein in solution at various pH values (20 mM Tris, pH 7.0, 50 mM NaCl; 20 mM sodium acetate, pH 5.0, 50 mM NaCl; 20 mM sodium citrate, pH 3.0, 50 mM NaCl; 20 mM glycine, pH 10.0, 50 mM NaCl) and its behaviour in the presence of aliphatic sulphonates was monitored by following the intrinsic fluorescence of the tryptophan residues using an Aminco BOWMAN series 2 spectrofluorometer. The excitation and emission bandwidths were 4 and 8 nm, respectively. The fluorescence cell (1×1 cm) was mounted on a thermostatic holder. Tryptophan fluorescence was measured at an excitation wavelength of 295 nm, and emission spectra were recorded between 340 and 420 nm. All measurements were performed using 10 µM protein and 20 µM sulphonates in 20 mM Tris, pH 8.0. Circular dichroism measurements were carried out on a JASCO J-810 spectropolarimeter equipped with a Peltier-type temperature controller and a thermostatic cell holder interfaced with a thermostatic bath. Spectra were recorded in quartz cells with a 0.1 cm path length at a protein concentration of 10 µM in various buffers. Twenty consecutive scans were accumulated and averaged. The data were corrected for the baseline contribution of the buffer, and the observed ellipticity was converted into the mean residue ellipticity [θ] based on a mean residue molecular mass of 34,000 Da. Secondary structure was estimated from fitted Far-UV CD spectra using the DICROPROT software package [32]. Thermal shift assays were performed with the dye SYPRO orange and conducted in the iCycler iQ Real Time Detection System (Bio-Rad, Hercules, CA). The T*m* represents the temperature at the midpoint of the unfolding transition. Various concentrations of protein, ligand and buffer were evaluated in 96-well iCycler iQ PCR plates for determination of the appropriate conditions for the assay. For the final experiments, solutions of 100 µl were prepared with 6 µM SsuA, 2.7X SYPRO orange, and 5 mM ligand in 20 mM Tris, pH 7.4, 50 mM NaCl. The plate was heated from 25°C to 85°C at a heating rate of 0.5°C/min. The fluorescence intensity was measured using excitation and emission wavelengths of 490 and 530 nm, respectively. Stock solutions of the alkanesulphonates tested were prepared at concentrations of 0.1 M and included HEPES [4-(2-hydroxyethyl)-1-piperazine ethane sulphonic acid] pH 7.0, MOPS (3-morpholinopropane-1-sulphonic acid) pH 6.9, MES [2-(n-morpholino)-ethanesulphonic acid] pH 4.5, CHES [2-(n-cyclohexylamino)-ethanesulphonic acid] pH 4.5, CAPS (N-cyclohexyl-3 aminopropanesulphonic acid) pH 4.5, PIPES [piperazine-n,np-bis(2-ethanesulphonic acid)], thiosulphate, pyridinium p-toluene sulphonate, 3-amino-1-propane sulphonic acid 97%, 3-hydroxylamine-O-sulphonic acid, 3-hydroxypropane-1-sulphonic acid and taurine.

**Crystallisation of the** ***X. citri*** **SsuA protein.** Crystallisation conditions were screened using sparse-matrix screens in 96-well plates with protein at a concentration of 6 mg/ml in 20 mM Tris buffer, pH 7.0, containing 50 mM NaCl at 18°C. SsuA crystallisation was performed with the sitting-drop vapour-diffusion method by mixing equal volumes (3 µl) of protein solution and crystallisation solutions. Data from the first crystals obtained in HEPES were collected at 100 K at the D03B-MX1 beam line Brazilian Synchrotron Light Laboratory (LNLS) [33] using 1.433 Å radiation and recorded on a *MARCCD*165 detector (oscillation data with Δφ = 1.0^o^). The crystals were cooled to 110 K in a stream of nitrogen gas to minimise radiation damage. Cryoprotection was obtained by soaking crystals into drops of crystal buffer plus 10% glycerol. Data collection was performed at 100 K at the D03B-MX2 beam line Brazilian Synchrotron Light Laboratory (LNLS) using 1.6 Å radiation and recorded on a *MARCCD*325 detector (oscillation data with Δφ = 1.0^o^).

**Structure solution, model building and refinement.** Diffraction data were indexed using the XDS package [34] and processed using SCALA and TRUNCATE [35]. NaI and CsCl_3_ derivative crystals were obtained by quick cryo-soaking for 15 s in a solution composed of the mother liquor supplemented with 0.5 M NaI or CsCl_3_ and 20% glycerol, yielding a data resolution of 2.4 Å. The SsuA 3D structure was solved by multiple isomorphous replacement and anomalous scattering (MIRAS) using the NaI and CsCl_3_ derivative datasets. The program AutoSHARP [36] was used to calculate the phases and to determine the incorporation of iodine and caesium chloride sites using SHELX [37] and density modification procedures. The ArpwArp [38] program was used to automatically build approximately 80% of the residues. Construction of the model was performed using COOT, and 14 cycles of refinement were realised using REFMAC [39]. The SsuA structure in the presence of MOPS and MES was obtained by molecular replacement using the structural coordinates of SsuA bound to HEPES (PDB code 3E4R).

**Construction of the** ***Xac::ssuA*** **mutant.** Site-specific inactivation of the *ssuA* gene was carried out with the *X. citri* 306 strain using a suicide plasmid carrying an inactivated copy of the *ssuA* gene for the selection of a gene replacement event, according to previously published procedures [40]. The first step involved cloning of the *ssuA* gene into the pUC4 plasmid after PCR amplification with primers FssuA2Nde28a (5′ GCGCATATGGCCGAGCCGGCGCA 3′) and RssuA2Hind28a (5′GCGAAGCTTTCATTTGCTCACC 3′) (the *Nde*I and *Not*I restriction sites are underlined). Inactivation of the *ssuA* gene was achieved by cloning a 2 kb gene cassette derived from *Sma*I-cleaved pHP45omega and encoding resistance to spectinomycin/streptomycin [41] into the single *Kpn*I cleavage site within the *ssuA* gene. A clone carrying an inactivated copy of the *ssuA* gene (*ssuA*::sp/sm) was selected and confirmed by restriction analysis and automatic DNA sequencing. The mutated copy of the *ssuA* gene was released for the next cloning step by cleavage with *Nde*I and *Not*I restriction enzymes. The mutated *ssuA*::sp/sm gene was cloned into the suicide pNPTS138 vector (originally constructed by D. Alley and kindly supplied by Dr. M. V. Marques of the Department of Microbiology, University of São Paulo). The pNPTS138 plasmid permits selection of single chromosomal integrations after plating transformed cells on plates containing kanamycin and subsequent selection of gene replacement events after a second selection round in media containing sucrose (because the plasmid carries a *sacB* gene that confers sensitivity to sucrose) and spectinomycin [41]. The recombinant plasmid was named pNssuA and introduced by electroporation into the *X.citri* 306 strain as previously described [40]. Initial selection for the gene replacement event was carried out on plates containing kanamycin, followed by overnight growth of selected colonies in nonselective media and subsequent plating on 3% sucrose and 50 µ/ml spectinomycin to screen for cells that had undergone a second crossover event leading to excision of the plasmid carrying the wild type *ssuA* copy. PCR with primers FssuA2Nde28a and RssuA2Hind28a was used to confirm chromosomal deletions.

**Construction of the expression vector for complementation of the** ***Xac::ssuA*** **mutant**. A fragment containing a 900 base pairs upstream of the XAC3200 gene from *ssu* operon was amplified by PCR using primers FssuSalI-p (5′ CCAGAAACGCATTCATGACCTC 3′) and RssuEcoRI-p (5′ CAGCCCATACAAAAGGACGTCA 3′). This fragment contained the promotor sequence, which was cloned into the pKX33 vector [42] for construction of the pKX33-p. The full *ssuA* gene (1026 bp) was amplified from *X. citri* genomic DNA by PCR using the primers FssuA-EcoRIc (5′ GAATTCATGCGGGCAACGGGCAGG 3′) e RssuA-XbaIc (5′ TCTAGATCATTTGCTCACCGCCTGCG 3′) and cloned in to the pKX33 with the promoter sequence to build the pKX33-p*ssuA* vector for expression in *X. citri* cells. The final plasmid was confirmed after digestion with *Sal*I and *Xba*I (**Figure 6C**) and automatic DNA sequencing. Competent cells of *Xac::ssuA* strain were transformed with the final vector pKX33-p*ssuA* by electroporation followed by selection on LB plates with 50 µg/ml kanamycin. The cells are described as *Xac::ssuAc*.

**Xanthan gum production.** *X. citri* 306 strain, the isogenic *Xac::ssuA* mutant and the mutant complemented (*Xac::ssuAc*) were grown in LB medium for 24 h at 30°C under aerated conditions (200 rpm) and subjected to the protocol described by Vojnov and collaborators [14]. Kanamycin (50 µg/ml) was added to the cultures of the mutant and complementary strains.

**Growth curves and plant infection experiments.** In vitro growth of wild-type and mutant strains was performed in 10 ml M9 minimal medium and sulphur-free M9 minimal medium supplemented with different alkanesulphonates (MES, CHES, HEPES) as sulphur sources at 28°C. Samples were taken every 2 h for determination of the total number of viable cells. In vivo assays were performed as previously described [43]. Six-month-old plants of sweet orange (*C. sinensis*) were obtained from certified nurseries and kept in a growth room at 25–28°C with fluorescent light illumination. Leaf sectors were infiltrated with approximately 7×10^6^ viable bacteria in a total volume of 0.3 ml. Bacterial cultures were prepared in LB without NaCl for 48 h at 28°C with shaking at 200 rpm and subsequently suspended in sterile water. The appearance of canker pustules and lesion phenotypes was monitored daily for 20 days. To follow the growth of the bacteria inside the plant tissue, leaves were infiltrated with 7×10^6^ viable bacteria in 6 different sectors. Every other day, three circular sections 1 cm in diameter containing the bacterial infiltrates were macerated in water and diluted aliquots were plated on LB agar plates for determination of the number of viable bacteria.

## Supporting Information

Table S1 **Crystallisation conditions for** ***X. citri*** **SsuA.**
(DOCX)

Table S2 **Ligand interactions formed by hydrogen bonds between** ***X. citri*** **SsuA and alkanesulphonates.**
(DOCX)
