# Supplementary material for: Structural and Physiological Analyses of the Alkanesulphonate-Binding Protein (SsuA) of the Citrus Pathogen Xanthomonas citri
Source: PLoS One. 2013 Nov 25;8(11):e80083. doi: 10.1371/journal.pone.0080083 (PMC3839906; doi:10.1371/journal.pone.0080083)
Supplement: Table S1 — Crystallisation conditions for X. citri SsuA. (DOCX) [file pone.0080083.s001.docx]

**Table S1. Crystallization conditions of *X. citri* SsuA.**

| **Condition** | **Salt** | **Buffer** | **Precipitant** | **Diffraction (Å)** |  |
| --- | --- | --- | --- | --- | --- |
| 1 | 0.2 M ammonium sulfate | 0.1 M MES pH 6.5 | 30% PEG MME 5000 | 4.8 |  |
| 2 | 2 M ammonium sulfate | 0.1 M HEPES  pH 7.5 | 2% PEG 400 | 4.0 |  |
| 3 | 0.2 M ammonium sulfate | 0.1 M MES pH 6.5 | 30% PEG MME 5000 | 3.8 |  |
| 4 | 2 M ammonium sulfate | 0.1 MES pH 6.5 | 5% PEG 400 | 3.6 |  |
| 5 | 2 M ammonium sulfate | 0.1 M HEPES pH 6.5 | 2% PEG 400 | 3.4 |  |
| 6 | 1.6 M ammonium sulfate | 0.1 M MES pH 6.5 | 10% diane | 2.1 |  |
| 7 | 0.1 M NaCl; 1.6 M ammonium sulfate | 0.1 M HEPES pH 7.0 | - | 2.1 |  |
| 8 | 0.1 M NaCl; 1.6 M ammonium sulfate | 0.1 M HEPES pH 7.3 | - | 1.9 |  |
| 9 | 0.1 M NaCl; 1.6 M ammonium sulfate | 0.1 M HEPES pH 7.5 | - | 1.7 |  |
