# Supplementary material for: Structural and Physiological Analyses of the Alkanesulphonate-Binding Protein (SsuA) of the Citrus Pathogen Xanthomonas citri
Source: PLoS One. 2013 Nov 25;8(11):e80083. doi: 10.1371/journal.pone.0080083 (PMC3839906; doi:10.1371/journal.pone.0080083)
Supplement: Table S2 — Ligand interactions formed by hydrogen bonds between X. citri SsuA and alkanesulphonates. (DOCX) [file pone.0080083.s002.docx]

**Table S2. Ligand interactions formed by hydrogen bonds between *X. citri* SsuA and alkanesulfonates.**

| **Residue in**  **SsuA** | **Atom in the ligand** | **Distance (Å)** |
| --- | --- | --- |
|  | **Hepes** |  |
| Gln36/NE2 | O1S | 2.75 |
| Gly68/N | O3S | 2.80 |
| Gly86/N | O1S | 2.90 |
| Gly86/N | O2S | 3.50 |
| Ser141/OG | O2S | 3.59 |
| Ser141/N | O2S | 2.90 |
| Asp186/N | O8 | 3.32 |
| Asp186/O | O8 | 3.46 |
| W106 | N1 | 2.68 |
| W117 | O8 | 3.01 |
| W118 | O8 | 2.71 |
| W118 | N4 | 2.76 |
|  | **MOPS** |  |
| Gln36/NE2 | O1 | 2.90 |
| Gly68/N | 02 | 2.80 |
| Gly86/N | O3 | 2.57 |
| Ser141/OG | O3 | 3.5 |
| Ser141/N | O3 | 2.60 |
| Thr109/OG1 | O4 | 2.90 |
| W001 | N1 | 2.90 |
|  | **MES** |  |
| Gln36/NE2 | O3S | 2.80 |
| Gly68N | O1S | 3.00 |
| Gly68N | O3S | 3.20 |
| Gly86/N | O3S | 2.90 |
| Ser141/OG | O2S | 3.20 |
| Ser141/N | O2S | 2.90 |
| W102/O | O4 | 3.00 |
